# Bioactive Natural Pigments’ Extraction, Isolation, and Stability in Food Applications

**DOI:** 10.3390/molecules28031200

**Published:** 2023-01-26

**Authors:** Adriana K. Molina, Rúbia C. G. Corrêa, Miguel A. Prieto, Carla Pereira, Lillian Barros

**Affiliations:** 1 Centro de Investigação de Montanha (CIMO), Instituto Politécnico de Bragança, Campus de Santa Apolónia, 5300-253 Bragança, Portugal; 2 Laboratório Associado para a Sustentabilidade e Tecnologia em Regiões de Montanha (SusTEC), Instituto Politécnico de Bragança, Campus de Santa Apolónia, 5300-253 Bragança, Portugal; 3 Grupo de Nutrição e Bromatologia, Faculdade de Ciência e Tecnologia de Alimentos, Universidade de Vigo, 36310 Vigo, Spain; 4 Programa de Pós-Graduação em Tecnologias Limpas, Instituto Cesumar de Ciência, Tecnologia e Inovação—ICETI, Universidade Cesumar—UNICESUMAR, Maringá 87050-390, Brazil

**Keywords:** anthocyanins, carotenoids, chlorophyl, colorants, extraction, stability

## Abstract

Color in food has multiple effects on consumers, since this parameter is related to the quality of a product, its freshness, and even its nutrient content. Each food has a characteristic color; however, this can be affected by the technological treatments that are applied during its manufacturing process, as well as its storage. Therefore, the development of new food products should take into account consumer preferences, the physical properties of a product, food safety standards, the economy, and applications of technology. With all of this, the use of food additives, such as dyes, is increasingly important due to the interest in the natural coloring of foods, strict regulatory pressure, problems with the toxicity of synthetic food colors, and the need for globally approved colors, in addition to current food market trends that focus on the consumption of healthy, organic, and natural products. It is for this reason that there is a growing demand for natural pigments that drives the food industry to seek or improve extraction techniques, as well as to study different stability processes, considering their interactions with the food matrix, in order to meet the needs and expectations of consumers.

## 1. Importance of Natural Colorant Application in the Food Industry

Color is an influential and crucial sensory parameter when accepting or rejecting a food product, as it creates an idea of the state and composition of foodstuff [1,2]. It is even the case that color can reduce the desire to eat or drink a certain food, as this attribute relates to other sensorial perceptions such as taste, smell, texture, or quality index. Thereupon, the food industry seeks technologies that guarantee a stable color during production, distribution, and storage processes, such that food products have the quality expected by consumers [2,3]. The European Food Safety Authority (EFSA—www.efsa.europa.eu, accessed on 25 November 2022) defines “food colors” as “food additives which are added to food aiming to make up for color losses following exposure to light, air, moisture and variation in temperature, to enhance naturally occurring colors and to add color to foods that would otherwise be colorless or colored differently” [4].

Food colorants have been employed since ancient times; the Romans and Egyptians used colorants to improve the appearance of food. In the Middle Ages, extracts of carrot, chard, or herbs were added to preparations to avoid food monotony [5]. At the beginning of the fourteenth century, with advancements in chemistry, foods were colored with different mineral salts, such as lead chromate, mercury sulfite, copper arsenate, or coal tar. The chemist William Perkin (1838–1907) developed the first synthetic organic colorant “aniline purple”, or “mauve”, from coal tar, which triggered the development of a wide variety of organic colorants, replacing mineral salts [6]. At the end of the century, some evidence of toxicity created demand for determining the safety of food additives. In 1883, Harvey W. Wiley presented the first law on colorants, and between 1916 and 1929 the use of 10 synthetic colorants (Red 2, Red 3, Red 4, Blue 1, Blue 2, Green 3, Yellow 5, and Yellow 6) by the food industry was regulated; however, in 1969 Soviet science concluded that the long-term ingestion of Red 2 had caused cancer in laboratory animals. This being the case, 1976 the FDA replaced Red 2 and Red 4 with the Red 40 colorant (Alluna Red AC), which was safer for use in food and other industries [6,7].

In the past decade, the European Food Safety Authority (EFSA) has been evaluating a large quantity of research associating the use of certain synthetic food colorants with increased hyperactivity in children [8], allergies, toxicity, attention deficit hyperactivity disorder (ADHD) in children [9], and carcinogenicity [10], among other health issues [11]. Therefore, in recent years there has been a growing interest in natural food dyes that not only hold the ability to pigment, but that also provide therapeutic outcomes to the consumers [12].

**Figure 1:**
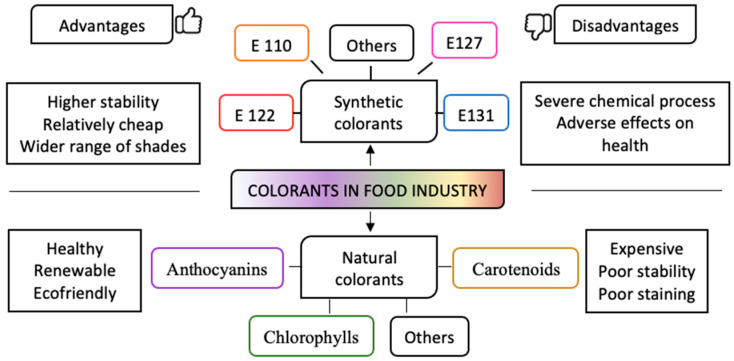
Natural and synthetic colorants’ advantages and disadvantages.

Currently, there are studies analyzing the different advantages and disadvantages of using natural and synthetic colorants. Synthetic pigments are more stable and less expensive; however, they can cause potential health problems, with allergic reactions, attention deficit in children, and cancer pointed out as the most common consequences [9,10]. Therefore, the study and development of natural colorants are stronger due to the benefits that they can provide to a consumer, since they have different compounds to which the coloration is attributed, besides providing antioxidant and antimicrobial bioactivities, among others [13]. Nevertheless, these pigments have several stability problems: they are sensitive to various external factors such as light, pH, and temperature, among others (Figure 1) [14]. In addition, only a limited number of natural dyes are available for use as food additives due to strict FDA and European Union restrictions as well as the requirement of rigorous safety evaluations for approval, which slow down the progress in this field of research [15,16,17].

This report brings together the most recent advances and challenges in this field, highlighting natural pigments for their diverse properties. The molecules considered in this report were anthocyanins, chlorophylls, and carotenoids, describing their chemical structures, the factors that affect their stability (pH, temperature, oxygen, etc.), their therapeutic effects, such as the reduction in coronary diseases, anticancer, antitumor, anti-inflammatory, and antidiabetic properties, the different classical as well as novel extraction methodologies, and the most commonly used purification methods according to the characteristics of each pigment. Additionally, a compilation was made of the formulations that are currently being studied to obtain natural colorants that are stable during the manufacturing, production, and storage processes of different food products. The aim is to contribute with information that can be useful for industries, researchers, and professionals of food science as well as nutrition to continue with the study, development, and application of natural colorants in the food industry.

## 2. Molecular Structures of Natural Origin with Colorant Properties

**Figure 2:**
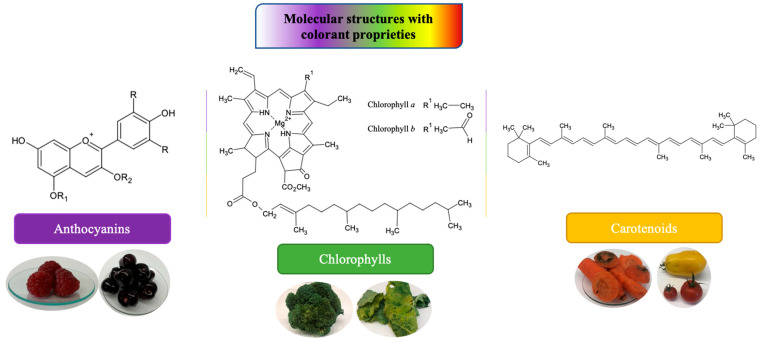
Molecular structures of representative food colorants from natural sources.

Natural colorants are selectively extracted from natural matrices such as plants, animals, or mineral substances. These are considered safe, so their use has fewer limitations than that of synthetic colorants. Natural pigments can be divided into three main groups: The first is heterocyclic compounds that contain oxygen as flavonoids (anthocyanins), these being found exclusively in vegetables and fruits. The second group includes heterocyclic compounds with a tetra-pyrrole structure, called chlorophylls, which are mainly present in vegetables. The third group has an isoprene structure, which represents carotenoid compounds that are mainly found in vegetables, algae, and bacteria, and that are ingested by animals in their diets (Figure 2) [3,18]. There is another group of pigments called betalains, which are water-soluble nitrogenous compounds derived from betalamic acid, mainly present in beets, that are responsible for a wide range of colorations, ranging from yellow to deep red, and are characterized by a high antioxidant capacity [19]. Curcumin is another natural colorant from turmeric (*Curcuma longa* L.) that is a diarylheptanoid belonging to the curcuminoid group. It imparts a very bright color; however, it is known for its low light stability with high water activity. Therefore, the most popular applications tend to be those with low water activity, such as high-boiling-point candies, jellies, and gum confectionery. It has been traditionally used as an antidiabetic and has been shown to possess anticancer and antioxidant properties [20]. On the other hand, spirulina also has the ability to provide an intense blue tone to food, besides having potential as an anticancer, antiviral, antioxidant, and antiallergic agent, which explains its increasing use. It is currently applied in foods with low water activity at neutral or slightly acidic pH values (pH > 4.5), such as candies, chewing gum, sugar decorations, sweets, dairy products, and ice cream. Its performance is poor under conditions that lead to the denaturation of the protein pigment, e.g., at pH values below 4.5 or at ethanol contents above 20% [21].

There are also pigments of animal origin; for example, the colorant carmin or carmic acid, known for its red color. It is obtained by drying and crushing female Cochineal insects (*Dactylopius coccus* Costa) and has a greater stability to exposure to high temperatures, light, and oxygen, and can be chelated with metal ions, forming carmine, compared to other natural dyes. Another dye that can be obtained from insects is Kermes, which is obtained from the adult female *Kermes ilicis* or *kermococcus vermilis* that lives in the young branches of the Mediterranean kermes oak (*Quercus coccifera* L.). Likewise, a bright red and scarlet dye can be obtained from a small red arthropod with dark legs called *Laccifer lacca* that lives on *Zyziphus Mauritania, Schleichera oleosa,* and *Butea monosperma* [22].

*Anthocyanins:* Anthocyanins enclose the most important group of water-soluble pigments, and they are detectable in the human visible region [23]. Found in the form of glycosides of anthocyanins, which consist of an aglycone (anthocyanidin) bound to some sugar by means of a glyosidic bond (Figure 2a) [24], these molecules are responsible for the different colors of vegetables, such as blueberries, blackberries, purple cabbage, haskap, etc., which have shades ranging from red to blue [25,26]. Color depends on several factors, such as chemical substituents in the general structure and the positions of these in the flavylium group; therefore, if phenolic rings have more hydroxyl groups, the blue color prevails, whereas the presence of methoxy groups results in a red tonality [27].

Beyond coloring properties, these molecules also present important beneficial effects in human health, such as reducing blood pressure, oxidative stress, and lipid peroxidation, as demonstrated in spontaneously hypertensive rats by cranberry anthocyanins [28]. Another study conducted in 2019 highlighted the potential of elderberry anthocyanins to protect cells from oxidative damage, measured by ROS, being in turn an alternative agent to modulate mitochondrial dysfunctions [29]. Regarding the anti-inflammatory capacity of anthocyanins, several studies have shown a reduction in proinflammatory bacteria populations in the intestinal microbiota due to the anthocyanins present in strawberry, blackberry [30], blueberry [31], and siyah goji [32]; however, these molecules present various stability problems due to different factors:

Effect of pH: The reversible transformations caused in anthocyanins by variations in pH make this parameter extremely important in their stability [3]. Thus, in aqueous solutions with a pH below 2, the pigment is red and very stable, since the predominant form is the flavylium ion; however, when the pH becomes alkaline, the flavylium ion undergoes nucleophilic attack by water, which produces the pseudo-carbinol base at a pH of 4.5, followed by the formation of chalcones that are colorless and very unstable. At pH values above 8, purple quinoidal forms occur, which are rapidly degraded through oxidation with air [33,34].

Effect of temperature: Anthocyanins lose their color when there is an increase in temperature (>25 °C) because the equilibrium between the structures is endothermic, which will cause two mechanisms to occur: hydrolysis of the glycosidic bond that leads to the formation of an aglycone, or hydrolytic cleavage that originates a chalcone. These can occur during the processing or storage of anthocyanins, compromising their stability [34].

Effect of light: Exposure to UV, visible light, or other sources of ionizing radiation makes anthocyanins generally unstable, mainly those that present substituents on the hydroxyl of carbon 5 [35].

Effect of oxygen: The presence of oxygen is a factor contributing to the degradation of anthocyanins, even in the absence of light. This occurs due to the direct or indirect oxidation of the medium components, with which they react. Color stability is improved when oxygen is excluded from the system by heating, a vacuum, or nitrogen flow, and a low pH is maintained because a high pH causes further degradation [36].

*Chlorophylls:* Chlorophylls are the most abundant natural pigments found in plants; they are responsible for photosynthesis [37]. They are complex molecules belonging to the class of porphyrins, formed by four pyrrole rings and a fifth isocyclic ring located next to the third pyrrole ring. The rings are interconnected by methylene bridges, and the molecule holds a magnesium atom inside. In the fourth pyrrole ring, the propionic acid present there is esterified by a long-chain acyclic alcohol, generally a phytol, which gives chlorophyll a hydrophobic character (Figure 2b) [38].

There are different types of chlorophyll: type *a*, present in most vegetables, is responsible for absorbing light during photosynthesis and nowadays is widely used as a natural colorant in the pharmaceutical and food industries; type *b*, located in chloroplasts, absorbs light of another wavelength and transfers energy for chlorophyll *a*; type c, which is found in the chloroplasts of brown algae; and finally, type *d*, which is only found in red algae [12,39]. Chlorophylls *a* and *b* are related to therapeutical effects, acting as anticancer, antibacterial, antioxidant, anti-inflammatory, and energizer agents; likewise, they improve the oxygenation of blood and the detoxification of the body [12,40]. In 2021, the contents of chlorophylls *a* and *b* in grapefruit leaves were studied, as was the ability of chlorophyll extract to inhibit the growth of A375 melanoma cells [41]. A significant decrease in glucose levels in diabetic mice was evidenced, showing the ability of *Sauropus androgynus* leaves to ameliorate the oxidative stress associated with diabetes mellitus [42].

The stability of chlorophylls is low, since their structure can be modified by different factors that consequently alter their chromatic properties. The most frequent reaction that affects the stability of chlorophylls is the substitution of the central magnesium ion by two hydrogens, generating a drastic change in color, since magnesium derivatives are green, while derivatives without magnesium (mainly pheophytins and pheorphorbides) are brown. Among the most relevant factors that alter chlorophyll color, photo-oxidation, heating, an acid medium, and freezing during storage are the most important, especially the latter factor, since low temperatures increase the tendency for the precipitation of food proteins by reducing the pH, which increases the speed of acid catalytic reactions, such as pheophytinization, thus influencing the stability of chlorophylls [43].

*Carotenoids:* Natural fat-soluble colorants with nutritional and antioxidant proprieties, carotenoids are responsible for the yellow, orange, and red colors of higher plants. Especially present in leaves, flowers, and fruits, they can be synthesized by plants, algae, and photosynthetic bacteria. Their structure belongs to the terpene family, consisting of eight units of isoprene that originate a skeleton of 40 carbon atoms. Carotenoids are classified in two large groups: carotenes, which are exclusively hydrocarbons, such as lycopene and β-carotene (Figure 2c), and xanthophylls, derived from the above by incorporating oxygenated functions as hydroxyl, methoxy, carboxyl, keto, or epoxy groups, such as lutein, β-cryptoxanthin, zeaxanthin, and fucoxanthin. Additionally, carotenoids may have an acyclic structure, such as lycopene, or possess different cyclic structure at one or both ends, such as β-carotene. Due to the large number of double bonds in the chain, carotenoids can exist in different *cis*/*trans* conformations, although the most stable and therefore present in nature is the all-*trans* [44,45].

The nutritional importance of carotenoids is mainly due to the fact that some have provitamin A activity [46,47]; however, various authors have reported these molecules as being antioxidant compounds [48,49] and beneficial for the prevention of various diseases, such as certain types of cancer [50,51], eye [52,53] and vascular disorders [54,55], and others. They have been found in tomatoes (*Solanum lycopersicum*), and an association between the antioxidant activity of lycopene and protection against the appearance as well as development of malignant or cancerous cells in the prostate was suggested [56]. Astaxanthin, the pigment with the highest antioxidant propensity, is not only found in vegetables and some species of freshwater microalgae (*Haematococcus pluvialis*); it is also found in some animal species, such as trout, salmonids, shrimp, and some species of mussels. Carotenoids, in particular astaxanthin, improve the response of the immune system and are also powerful anti-inflammatories; therefore, in addition to preventing aging, they are very beneficial in most pathologies with chronic levels of inflammation, such as arthritis, muscle pain, cardiovascular disease, and Alzheimer’s disease, also being known to regulate cholesterol levels and contribute to good blood circulation [57].

The stability of carotenoids is mainly related to the large number of double bonds of their molecules, which makes them vulnerable to oxidative processes, especially in photo-oxidation reactions with singlet oxygen. Carotenoids also oxidize in the presence of lipoxygenases, but not directly, still via reacting with hydroperoxides. Hence, exposure to light, temperature, or pH leads to color loss and a consequent decrease in food nutritional value [58]. Table 1 shows different studies of natural matrices rich in anthocyanins, chlorophylls, and carotenoids, with their respective properties and beneficial effects on consumer health.

## 3. Extraction and Purification of Plant-Based Extracts

When working with molecules from natural matrices, it is important to consider that compounds are found in very complex mixtures in nature. This being the case, in order to obtain a natural pigment in the purest possible way, different methods of extraction and purification should be employed and depend on the nature and further application of target molecules. A crucial step is determining the best extractor solution, since it must be able to extract the metabolites of interest, be easy to remove, should not react with the matrix, and, when making pigments for the feed area, cannot be toxic. It is also important to consider particle size, porosity, and temperature, since many metabolites are thermolabile. Considering the above, there is a demand for the optimization and standardization of recovery approaches that not only ensure recovered compounds’ functionality and maximum recovery yields, but also meet principles of green chemistry and sustainability [80,81,82,83].

Different extraction methods are developed that not only produce high yields of anthocyanins, chlorophylls, and/or carotenoids, but also consider the conditions that influence the stability of such molecules, namely temperature, pH, time, extractor solvent, and concentration, to avoid loss of functionality [82]. Likewise, the use of simple, fast, and low-cost methods, the use of solvents of low or no toxicity, and their application in industries are important factors when choosing an optimal extraction method [84].

On the other hand, concerning the methods of separation of compounds and their identification, the application of an adequate and efficient purification process to the extract avoids extra costs to the process, in addition to minimizing the loss of solvents and reagents. Currently, chromatography is one of the most widely used methods, being considered as more accurate than spectrophotometry and fluorimetry [85,86,87].

### 3.1. Anthocyanins

Anthocyanins are polar molecules due to the aromatic rings with substituent groups (hydroxyl, carboxyl, and methoxyl) as well as glucoside residues within their structure. They are traditionally extracted from plants using methanol acidified with hydrochloric acid or formic acid. Acidification is performed because low pH values prevent the degradation of non-acylated anthocyanin pigments; however, the food industry has preferred other extractor solutions due to the potential toxicity of traditional ones [88,89].

In anthocyanin extraction, the classical method is solid–liquid due to the polarity of these molecules in solvents such as methanol/ethanol and acetone, which must be acidified; however, new approaches based on clean technologies have been developed to improve anthocyanins’ extraction yields, such as enzyme-assisted extraction, supercritical fluid extraction, ultrasound-assisted extraction, and pressurized liquid extraction (also known as solvent-accelerated extraction), alongside microwave-assisted extraction, ohmic heating-assisted extraction (synonymous with electroconductive heating), and others [90]. In 2013, the process of extraction via the maceration of eggplant (*Solanum melongera* L.) pulp and peel was optimized by using response surface methodology, considering three factors: solvent concentration, time, and temperature. The results show that the optimum extraction conditions are 50% solvent (ethanol), 4 h and 30 °C, with a content of 62 mg/100 g of anthocyanins [91]. Puertas et al. (2013) studied the anthocyanin content in beans (*Phaseolus vulgaris* L.), comparing solid–liquid extraction and microwave-assisted extraction (MAE). The authors verified that both techniques provided optimal results; however, the use of microwaves significantly reduced solvent use as well as the thermodegradation of the matrix and analytes [92]. Another study conducted by Flores et al. in 2017, with elderberries (*Sambucus nigra* L. subsp. Peruviana), evaluated the use of enzymes, ultrasound, microwaves, and maceration in order to find the most efficient extraction method, where maceration produced extracts with better antioxidant characteristics, followed by microwaves, enzymes, and ultrasound [93].

In a more recent study, Grillo et al. in 2020 studied two green extraction methodologies, namely microwave-assisted extraction (MAE) and ultrasound-assisted extraction (UAE) for the recovery of anthocyanins from mulberry residues, using five distinct natural deep eutectic solvents (NADES). Both technologies obtained superior performance in comparison with conventional extraction. MAE and EAU yielded 25.83 and 21.18 mg/g_matrix_ of total anthocyanin content, respectively, after 15 and 30 min of extraction [94]. In the same year, a study was carried out to optimize the recovery of anthocyanins from blackberry wine residues by employing ultrasound-assisted enzymatic extraction. The authors reported the identification of two anthocyanins (cyanidin-3-*O*-glucoside and cyanidin-3-*O*-ruthinoside) as the major compounds of the extracts, suggesting that this approach is efficient, economical, and environmentally friendly for anthocyanin recovery [95].

Other extraction methodologies for phenolic and anthocyanin recovery are extraction with supercritical fluids (SFE) and extraction with pressurized liquids (PLE), as they operate with low temperatures and short extraction times, avoiding the degradation of thermolabile secondary metabolites and allowing the use of non-toxic economic solvents, carbon dioxide being one of the most used [96]. In a study conducted in 2020, two optimized PLE methods were used for the extraction of anthocyanins and total phenolic compounds from açai berries. The percentage of methanol in the extraction solvent proved to be the most significant variable for anthocyanin extraction. The methods developed showed high precision, with relative standard deviations (RSDs) lower than 5% [97].

Enzyme extraction is another relatively novel technique that is still under development. One of the studies in which its efficacy was evidenced was conducted by Swer TL. et al. (2018). The authors recovered anthocyanins from *Prunus nepalensis* L. by using cellulase and reported the detection of cyanidin-3-*O*-glucoside, petunidin-3-*O*-glucoside, peonidin-3-*O*-glucoside, and malvidin, in addition to a higher recovery of anthocyanins in comparison with conventional solvent extraction process [98].

On the other hand, it has been evidenced that the preparation of juices allows different metabolites, such as anthocyanins, to be easily acquired, these being one of the most used to obtain natural colorants. Türkyılmaz et al. (2019), for instance, obtained juices from Guindas (*Prunus cerasus* L.) of the Kütahya variety with high anthocyanin concentrations (168 mg/L of cyandin-3-*O*-glucosylrutinoside and 62 mg/L of cyanidin-3-*O*-rutinoside) to perform a co-pigmentation analysis [99]. Likewise, Molina et al. (2019) made a juice from haskap (*Lonicera careulea* L.) fruits with high anthocyanin content, obtaining solid coloring formulations with antioxidant and antimicrobial properties for application in food [25]. Table 2 compiles different anthocyanin extraction studies and describes, in detail, the optimized method used in each investigation.

The different extraction methodologies may not be selective for anthocyanins; therefore, non-phenolic substances such as sugars, organic acids, and proteins may be present in the extract. It is therefore important to apply an adequate and efficient purification process, considering that 80% of process costs are associated with purification methods [100]. A great variety of techniques have been studied in order to obtain an extract free of any undesired component, ranging from solid-phase extractions (SPEs) and liquid–liquid extractions (LLEs) to the use of chromatographic techniques such as counter current chromatography [101,102], medium-pressure liquid chromatography (MPLC), UPLC, and HPLC. Currently, the most widely used method for the separation of anthocyanins is HPLC with UV–Vis or photodiode array detectors (PDA) [103,104].

**Table 2:** Comparison of different methods employed for anthocyanin extraction.

Plant Matrix	Extraction Aaproach	Solvent	Extraction Conditions	Anthocyanin Recovery Yield	Ref.
Purple sweet potatoes (*Ipomoea batatas* L.)	Conventional solvent extraction	Ethanol 80%; HCl 0.1% (*v*/*v*)	T (°C): 60 t (min): 90	217.58 mg·(100 g)^−1^ Cyanidin-3-*O*-glucoside DW	[105]
	Ultrasound-assisted extraction	Ethanol 90% (*v/v*); HCl 0.1% (*v*/*v*)	T (°C): 50 t (min): 45 Power (W): 200	229.41 mg·(100 g)^−1^ Cyanidin-3-*O*-glucoside DW	
	Accelerated solvent extraction	Ethanol 80% *v*/*v*; HCl 0.1% (*v*/*v*)	T (°C): 90 Static time (min): 15 Static cycle: 2	244.07 mg·(100 g)^−1^ Cyanidin-3-*O*-glucoside DW	
Blackberries (*Rubus glaucus* Beneth)	Cold extraction	Methanol; C_6_H_8_O_9_ 1%	t (h): 72	1.478 g·kg^−1^ Cyanidin-3-*O*-glucoside	[80]
Purple corncob (*Zea mays* L.)	Conventional solvent extraction	Ethanol 20%, pH of 2	T (°C): 25, 60, 75, and 90 t (min): 30, 60, 120, and 240 The best extraction conditions (75 °C and 240 min)	Values between 11.567 and 37.127 mg·g^−1^ of purple corncob Total anthocyanins	[106]
Eggplant (*Solanum melongena* L.)	Heat solvent extraction	Ethanol 50% *v*/*v*; orthophosphoric acid 1%	T (°C): 305 t (h): 4	62 mg·(100 g)^−1^ in eggplant peel Total anthocyanins	[91]
Haskap berry (*Lonicera caerulea* L.) pulp	Conventional solvent extraction	Methanol/water 80:20 (*v*:*v*); formic acid, 0.02 mL	T (°C): 35 t (min): 20	38.3% Total anthocyanins	[107]
	Supercritical carbon dioxide (scCO_2_)	Water	The highest total anthocyanin (TA) yield was achieved at 45 MPa, 65 °C, and 5.4 g water to 3.2 g berry pulp paste, 15 min static and 20 min dynamic time	52.7% Total anthocyanins	
Haskap berry (*L. caerulea*) pulp	Juice extraction	Water	Two-step press process followed by osmotic treatment	24.58 mg Cyanidin-3-glucoside/g DW	[108]
			One press and osmotic treatment	32.24 mg Cyanidin-3-glucoside/g DW	
Haskap berry (*L. caerulea*) pulp	Conventional solvent extraction	Ethanol/water 80:20 (*v*:*v*); trifluoroacetic acid 0.1%	Double extraction of 1 h each	97.9 mg·g^−1^ ext. Total anthocyanins	[25]
Blueberries (*Vaccinium* sp.), O’Neal variety	Solid–liquid extraction	Ethanol; citric acid 1%	T (°C): 36 T (h): 2	879.0 mg·(100 mL)^−1^ Cyanidin-3-glucoside	[82]
Mulberry (*Morus alba* L.) wine residues	Ultrasonic-assisted enzymatic extraction	Water acidified to a pH of 3.5 Enzyme dosage: 0.22%	T (°C): 52 Power (W): 315 t (min): 94	5.98 mg·g^−1^ Total anthocyanins	[95]
Açai (*Euterpe oleracea* Mart.)	Pressurized Liquid Extraction	Methanol/water 43%	T (°C): 81 200 atm 60 s purge pH: 7.00 50% flushing	5.76 mg·g^−1^ açai Total anthocyanins	[97]
Blueberry (*Vaccinium myrtillus* L.) peels	Microwave extractions	Natural deep eutectic solvent (Choline chloride:lactic acid)	T (°C): 60 T (min): 15	25.83 mg·g^−1^ matrix Total anthocyanins	[94]
	Ultrasound-assisted extractions	Natural deep eutectic solvent (Choline chloride:lactic acid)	30 min of sonication Power (W): 500	21.18 mg·g^−1^ matrix Total anthocyanins	
Residues of red grape (*Vitis vinifera* L.) skins	Ohmic heating effect	Water	I. T (°C): 40; t (min): 20 II. T (°C): 40 a 100; t (s): 20 Electric field: 80 and 16 V/cm Frequency (kHz): 25	1349 μg·g^−1^	[109]

### 3.2. Chlorophylls

Due to the strong absorption of chlorophylls in the electromagnetic spectrum between the blue and red regions, these pigments hold an intense green coloration [110]. In general, chlorophylls are unstable and sensitive to light, heating, oxygen, and chemical degradation, so it is necessary to study, from the different matrices, specific conditions with which to obtain chlorophyll extracts. In addition, variation sources that may interfere with the process, such as the nature, polarity, and purity of the solvent, temperature, and incubation time, as well as the methods used to identify and quantify chlorophylls must be considered [111]. Therefore, some of the methods described in the literature for the extraction as well as purification of chlorophylls from plants and green algae can be considered complicated, wasteful, or expensive processes.

In 1985, the extraction and purification of chlorophylls from plant sources were carried out using acetone and methanol as solvents due to the polarity and molecule–solvent interaction. During the process, the solution formed was cooled with liquid nitrogen and taken to reflux with filtration to perform the rupture as well as separation of the matrix elements. According to the author, the process is expensive and inefficient, considering the high rigor and complexity involved [112]; however, this method was complemented by the study of Lichtenthaler (1987), in which the extracted pigments were purified in a polyethylene chromatographic column by using the same solvent applied in extraction. Finally, the pigment fraction was separated by using sugar chromatographic columns and crystallized in the presence of iso-octane. The author reported that pure organic solvents gave better results for chlorophyll recovery in the laboratory, increasing the yield of the whole process [113]. In another study, maceration extraction was optimized with the use of different solvents, varying the different existing conditions (volume of solvent, with controlled temperature and humidity) for the extraction of chlorophylls *a*, *b,* and total (*a* + *b*) from the forage ramin Tifton 85 (*Cynodon* spp.), confirming that the use of organic solvents associated with destructive extraction methods gives good results [111]. Nonetheless, conditions such as the temperature, solvent/matrix ratio, and extraction time must be considered in order to evaluate and determine the best model for the extraction as well as quantification of chlorophyll from natural matrices [114,115,116,117]. According to D. Barnes et al. (1992), the use of dimethyl sulfoxide (DMSO) as the solvent, followed by repetitive washing with acetone, provides optimal results for chlorophyll extraction, avoiding the degradation of this molecule into pheophytin in addition to being hydroscopic and miscible in water, thus providing more agility in the process [118]. This information was corroborated and complemented by Tait, M.A. and Hik, D.S., who indicated that N,N-dimethylformamide (DMF) is effective for extractions that do not involve the destruction of the matrix and the use of maceration [85].

Other non-conventional methods are also studied for the extraction of chlorophylls in order to obtain better extraction results. Molina et al. (2022) extracted chlorophylls from the aerial parts of tomato and carrot, using maceration as well as ultrasound-assisted extractions and evaluating different parameters in each of them [119]. The ultrasound technique was more effective than maceration, where tomato aerial parts revealed a higher concentration of chlorophylls (211.6 ± 0.3 μg/g) than carrot aerial parts (110.4 ± 0.4 μg/g) did. In another study, maceration, Soxhlet, ultrasound-assisted, and pressurized liquid extractions were compared for the recovery of chlorophylls from the green microalgae *Chlorella vulgaris*; all techniques were optimized with a central composite design. Pressurized liquid extraction yielded the best results among the four investigated methods [120].

For the use of chromatography, attention must be paid to the selection of the stationary phase as well as to the elution program considering the polarity range, since in chlorophylls this is high, in addition to some of them having an acidic character. Generally, for the separation of chlorophyll derivatives, reversed-phase columns (C18 and C30) are the preferred stationary phase. Different mixtures of common organic solvents (methanol, acetone, and acetonitrile) and water are used in the mobile phase. Resolution is often improved when ion suppression or ion pairing techniques are used [121]. Table 3 describes different chlorophyll extraction studies, detailing the method optimized in each investigation and indicating the extraction, identification, and quantification of these compounds via different methods, matrices, conditions, and solvents. The purpose of these studies was the quantification and validation for the use of chlorophyll pigments as natural colorants and antioxidants in the food industry.

### 3.3. Carotenoids

Carotenoids are compounds of great interest at the industrial level, not only due to the colors that they impart (yellow, orange, and red), but also to the therapeutic properties that they provide to the consumer, such as reducing cancer, being a source of provitamin A, and helping to improve the immune system thanks to their antioxidant properties in addition to hepatoprotective and antibacterial effects [45,124].

Carotenoid extraction techniques should be selected with the aim of minimizing compound degradation, considering that environmental factors such as heat, light, and oxygen can affect the structure of these molecules, resulting in a low efficiency of the extraction process. Therefore, the extraction time, extraction solution, appropriate container, and temperature must be considered for an optimal extraction process [125].

As for the solvents, these must be organic due to the hydrophobicity that carotenoid molecules hold. For non-polar carotenoids, the most frequently used solvents are hexane and petroleum ether, whereas polar solvents such as acetone, ethanol, and methanol are suitable for the recovery of polar carotenoids [17]. To prevent oxidation, especially when it is not possible to work under an inert atmosphere or when the extraction process time is too long, antioxidants such as pyrogallol, sodium ascorbate, BHT, or ascorbyl palmitate are added to the extraction medium. Correspondingly, weak bases, such as calcium carbonate, magnesium carbonate, or sodium bicarbonate (1 g/10 g of sample), can be added to the extraction medium to neutralize the acids that are released. Another preventive measure with which to avoid the degradation of carotenoids is to work in cold conditions, adding dry ice or working with precooled solvents [124].

For the extraction of carotenoids, the classical extraction techniques are liquid–liquid extraction (LLE), solid–liquid extraction (SLE), or Soxhlet extraction; however, such methods present the following disadvantages that can affect the extraction yield: they require high amounts of organic solvents, are time-consuming, use high temperatures, and are laborious. Therefore, new extraction processes have been developed to obtain results that are equal or better to those of conventional techniques, yet which use less organic solvent, are of a higher selectivity, have reduced process times and temperatures, and consume less energy. Supercritical fluid extraction (SFE), pressurized liquid extraction (PLE), subcritical water extraction (SWE), microwave-assisted extraction (MAE), ultrasound-assisted extraction (UAE), and liquid-phase microextraction (LPME) are some of the advanced extraction techniques that have been applied in carotenoid extraction (Table 4) [125,126,127].

Within the techniques described above, the best conditions for carotenoid extraction have been studied. In 2019, the parameters of temperature, time, and solute–solvent ratio were evaluated to obtain carotenoids from dried palm peach peel using ultrasound-assisted extraction. The obtained data, analyzed with response surface methodology (RSM) and central composite design (DCP), gave the optimal conditions for carotenoid recovery: 48 °C, 28 min, and a solute–solvent ratio of 0.0037 g/mL. The total carotenoid content was 151.50 mg/100 g of sample, a result that was 33.60% higher than the one observed for the maceration technique [126]. In the same year, Tiwari et al. compared the ultrasonic and high-shear methods for recovering carotenoids from carrot pomace, using various combinations of time and temperature in addition to linseed oil as the solvent. In this study, the shear technique produced a higher content of carotenoids (94.8 ± 0.08%) for food applications [128]. Another group that used linseed oil as a solvent for obtaining carotenoids from carrot juice processing residues optimized microwave-assisted extraction (microwave power, extraction time, and oil/residue ratio) and compared this technique with conventional carotenoid extraction. The latter required 180 min to achieve a yield of 87%, while the microwave technique extracted about 78% in the first 9.39 min, revealing a considerable energy economy through the emergent extraction approach [129].

Likewise, the extraction of passion fruit rind with ethanol was assessed by using three techniques: immersion, thermostatic bath, and Soxhlet extraction, the latter providing the highest extraction yield where the parameters of ethanol concentration (between 80% and 90% *v/v*), solvent–raw material ratio (with ratios between 40:1 and 50:1), and time (defined between 90 and 150 min) were analyzed. The results were evaluated using the response surface model: the highest yield was achieved with 90% ethanol, 50 mL/g bark, and 150 min of operation, providing an extraction yield of 2208.53 μg β-carotene/100 g sample [130]. In 2019, the parameters of supercritical CO_2_ extraction of carotenoids from mango peel were optimized: the highest recovery yield of 1.9 mg of all-trans-carotene equivalent/g of dried mango peel was registered for 26 MPa, 60 °C, and 15% *w/w* ethanol [131]. Considering all of the above, the use of novel extraction techniques as alternatives to conventional extraction methods offers several advantages, from extraction efficiency to being environmentally friendly; however, it is necessary to continue studying and optimizing these techniques since many of them have a limited field of applications, which impairs their implementation in industrial-scale systems [83].

**Table 4:** Comparison of different methods studied for carotenoid recovery.

Plant Matrix	Extraction Approach	Solvent	Conditions	Carotenoid Recovery Yield	Reference
Pericarp of tamarillo (*Cyphomandra betacea* Sendt var. roja)	Conventional solvent extraction	n-Hexane/petroleum ether 50:50%	t (h): 48 Absence of light	0.051 g CT/g pericarp	[132]
Tomato (*Solanum lycopersicum* L.) byproducts	Soxhlet	Ethanol	t (h): 5	0.703 mg/g lycopene 0.034 mg/g β-carotene extract	[133]
Peach palm (*Bactris gasipaes* Kunth) fruit peel	Ultrasound-assisted extraction	Soybean oil	T (°C): 48 t (min): 28 Solid–solvent ratio (g/mL): 0.0037	151.50 mg/100 g of dry peel Carotenoid content	[126]
Enzyme-treated carrot (*Daucus carota* L.) pomace	Ultrasonication	Flaxseed oil (green solvent)	Cycle: 45% Probe radius: 13 mm Power (W): 750 t (min): 12	21.67 ± 0.40 μg/g Total carotenoid content	[128]
	High-shear dispersion	Flaxseed oil (green solvent)	20,000 rpm t (min): 12	82.66 ± 0.06 μg/g Total carotenoid content	
Passion fruit cortex (*Passiflora edulis* f. *flavicarpa*)	Immersion	Ethanol 90%, acidified with citric acid at 0.03%	T (°C): 29 t (h): 2 500 RPM No light	113.08 ± 8.84 μg of β-carotene/100 g	[130]
	Thermostatic bath	Ethanol 90%, acidified with citric acid at 0.03%	T (°C): 60 t (h): 24	10.34 ± 5.18 μg of β-carotene/100 g	
	Soxhlet	Raw material–solvent ratio: 1:40	t (h): 2	1037.99 ± 48.70 μg of β-carotene/100 g	
Cantaloupe melon fruits (*Cucumis melo* L.)	Ultrasound-assisted extraction	Hexane/acetone 80:20	Amplitude: 100% t (min): 10	124.61 ± 3.82 μg/g	[134]
Canistel (*Pouteria campechiana* Kunth) Baehni.) fruits	Agitation Extraction	n-Hexane Dichloromethane n-hexane/dichloromethane (1:1) Ratios of solvent to sample of 15:1	T (°C): 40 200 rpm t (min): 30 After 6000 rpm t (min): 10	5.17 ± 0.08 g β-carotene equivalent per 100 g dry weight	[135]
		n-Hexane Dichloromethane n-hexane/dichloromethane (1:1) Ratios of solvent to sample of 30:1	T (°C): 40 200 rpm t (min): 30 After 6000 rpm t (min): 10	3.12 ± 0.01 g β-carotene equivalent per 100 g dry weight	
Carrots (*Daucus carota* L.) peels	Supercritical CO_2_	Ethanol 15.5%	T (°C): 59 p (bar): 349	86.1% of carotenoid recovery	[136]
Carrot (*Daucus carota* L.) juice processing waste	Microwave-assisted extraction	Oil (8.06:1 g/g)	Power (W): 165 t (min): 9.39	77.48%	[129]
	Conventional extraction	Oil (20:1 g/g)	T (°C): 65 t (min): 30 and 180	50% and 87% of carotenoid recovery	
Mango (*Mangifera indica* L. var. Sugar) peel	Supercritical fluid extraction	Ethanol 15% *w*/*w*	25.0 MPa T (°C): 60	1.9 mg all-trans-β-carotene equivalent g^−1^ dried mango peel	[131]

## 4. Stabilization of Natural Colorant Formulations

Natural colorants derived from anthocyanins, carotenoids, and/or chlorophylls have low stability due to their sensitivity to factors such as light, pH, temperature, and oxygen, among others; therefore, these pigments may degrade during the extraction and storage processes [137]. In the food industry, there are different encapsulation processes to avoid such degradation, through which it is necessary to coat the active substance (core) with some coating material (encapsulant), thus obtaining various forms of capsules, such as films, spheres, or irregular particles, as well as various types of structures (porous, compact, amorphous, or crystalline). These capsules are able to release their contents under specific conditions, which allows for the better bioavailability and stability of the bioactive compounds. The success of each of these processes depends on the choice of encapsulating material, which must consider the physical and chemical properties of the active ingredient, the method by which it will be encapsulated, the particle size to be obtained, and its practical application [138,139].

The most widely used technique with which to encapsulate bioactive compounds in the food industry is spray drying. Despite being an old technique, used since 1950, and the different advances in other encapsulation methods, it is still one of the most economic methodologies, with high quality, yield, size, and stability of the capsules [138]. Spray drying allows for a wide variety of encapsulating agents, including polysaccharides, such as starches, inulin [140], maltodextrin [25], or dextrose [141], corn syrups, gum arabic [26], mesquite gum [142], lipids, such as stearic acid and mono- as well as diglycerides, and proteins, such as gelatin, casein, whey, soy, and wheat [143].

Likewise, freeze-drying is a process that is widely used in the food industry due to its simplicity, flexibility, and ease of scale-up. This process is suitable for encapsulating high-temperature sensitive compounds such as anthocyanins, chlorophylls, and carotenoids, and also helps to preserve most of the initial properties of the material to be encapsulated, such as shape, dimensions, appearance, flavor, color, texture, and biological activity [144]. For example, in 2016, phenolic extracts from grape skin (*Vitis labrusca* var. Bordo) were encapsulated using gum arabic and an inlet temperature of 140°C, thus ensuring the retention of phenols (81.4 to 95.3%), anthocyanins (80.8 to 99.6%), and antioxidant activity (45.4 to 83.7%) [145]. In another study, the effect of different drying methods (freeze-drying, vacuum drying, sun drying, and oven/hot air tray drying) on the antioxidant activity, antimicrobial activity, and color of *Camellia assamica* leaves was investigated. The authors reported that freeze-drying was significantly better in all of the parameters studied, with the best yields for chlorophylls *a* and *b* as well as higher antioxidant activity values [146].

Another encapsulation method is thermal gelation, in which the filler is conditioned within small droplets in an aqueous layer, wrapped in a gelled wall. It is a simple and low-cost method where different encapsulation agents have been used, such as pectins in soluble solutions at pHs between 2.8 and 3.5 [147]; calcium alginate, which has multiple applications in the food area as a stabilizing, gelling, thickening, and microencapsulating agent [148]; and curdlana gum, which has the ability to form two types of gels depending on the heating, one reversible (low-set) and the other irreversible (high-set), with different characteristics [149].

On the other hand, there is the emulsion process, where two immiscible liquids are mixed more or less homogeneously due to the presence of an emulsifier that serves as a point of union between the two liquids. This technique, in addition to serving in the manufacturing of food, has been used in recent years to produce encapsulation systems and the controlled release of bioactive compounds to protect interactions and/or degradations, improving their functionality and bioavailability [150]. There are different types of emulsion (single, double, or multiple), allowing rapid or controlled release. A study conducted in 2018, for instance, created a water-in-oil-in-water double emulsion system suitable for the co-encapsulation of phenols and anthocyanins from a blueberry pomace extract. High co-encapsulation rates of blueberry polyphenols and anthocyanins, around 80% or more, were achieved when the oil droplets were relatively small (mean diameter of < 400 nm), with low dispersity (< 0.25) and a high negative surface charge (−40 mV or less) [151]. Likewise, Petito et al. (2022) developed carotenoid-rich red bell pepper extract powder nanoparticles produced by emulsification followed by lyophilization with four different encapsulating agents: calcium caseinate (ECC), bovine gelatin (EBG), and whey protein isolate (EWPI) as well as concentrate (EWPC). The nanoformulations presented spherical shapes and a heterogeneous distribution profile, showing a carotenoid encapsulation efficiency of 54.0% (ECC), 57.6% (EWPI), 56.6% (EWPC), and 64.0% (EBG). As for the encapsulation technique employed, it effectively increased the dispersibility of carotenoids in water, indicating their potential to be applied as natural food pigments [152]. Another technique that involves the emulsification of the active material and the wall material through a die at high pressure is microencapsulation via extrusion, which consists of producing small droplets of the encapsulating material by forcing a solution through nozzles or small openings in droplet-generating devices. The smaller the inner diameter of the nozzle or apertures, the smaller the capsules; an advantage of extrusion technology is that, in most cases, a true encapsulation procedure is achieved, rather than simple immobilization [153].

Liposome encapsulation is a technique that forms vesicles by means of phospholipid layers. The rolling of the lipid layer into a spherical shape forms a stable capsule, as there is no interaction of lipids with water; the sphere varies in size, from a few nanometers to microns. This technique has multiple benefits, such as greater stability and the possibility of large-scale production using natural ingredients. Liposomes are widely used in the food industry, both in research and industrial processes. Liposome preparation methods include mechanical methods, such as extrusion, sonification, high-pressure homogenization, microfluidization, and colloidal milling, as well as non-mechanical methods, such as reverse-phase evaporation and the micellar depletion of detergent–lipid mixtures [154,155]. Table 5 presents the advantages and disadvantages of all of the stabilizing methods mentioned above. There are also some studies that include the encapsulation of natural pigments rich in anthocyanins, chlorophylls, and carotenoids.

## 5. Application of Colorant Formulations in Food

Essential in the food industry, food colors are able to produce effects such as the desire for the consumption or even the rejection of a foodstuff, and are also indicators of good or poor food quality, significantly affecting the acceptance of consumers and playing a vital role in the market of a product. Whether from a synthetic or natural origin, their intended functional application is to improve the appearance of foods and beverages, or to restore the color loss caused by food processing and transformation. Used in certain concentrations, these additives cannot impart food flavor, and their use in the food industry is mainly in confectionery, bakery, beverages, dairy products, and meat [171,172].

Despite the many advantages of synthetic colors, the discovery of some side effects and toxicity problems, as well as the active growth of the natural, organic, and sustainable food markets, have led to a greater demand for natural coloring agents, with several already approved for use by regulatory authorities. Anthocyanins, chlorophylls, and carotenoids are some examples of natural colors that are currently used, identified according to the numbering system used by the Codex Alimentarius Commission, with the codes E163, E140–E141, and E160a to E161b, respectively [171].

As far as color formulations of anthocyanins (E163) are concerned, they have been used in a wide variety of products, with their main application being in soft drinks (low pH), confectionery, fruit preparations, dairy products, such as cream cheese, fermented milk, and milkshakes, and in solid food matrices, such as pancakes and omelettes [173,174,175]. They are generally employed at quantum satis, except in breakfast cereals, where only a maximum amount of 200 mg/L or mg/kg is allowed, depending on the case [176]; however, restrictions on the use of anthocyanins in some food products vary between countries, with the US generally being the most restrictive country on the use of coloring additives [177].

Due mainly to their difficult stabilization, among the different existing chlorophylls only two are used in the food industry as colorants: chlorophylls *a* and *b* (E140); however, more stable copper complexes are also allowed (E141) [171]. The E140 dye includes direct derivatives of chlorophyll, E140i being the direct fat-soluble originator of chlorophyll obtained from plant extraction, whereas E140ii (chlorophyllin) is water-soluble and is produced via the saponification of the natural extract, having a slightly higher stability than chlorophyll [43]. Copper chlorophyll complexes (E140), on the other hand, have a higher stability as well as solubility than the aforementioned ones and are not considered harmful to health, since the copper ions are not released in the digestive tract. In the food industry, commercial formulations of colorants based on chlorophylls, chlorophyllin, and copper complexes are used in beverages, jams and jellies, candies, chewing gums, dairy products, confectionery, soup concentrates, spreads, and canned as well as pickled vegetables [178]. Still, the US FDA only authorizes the use of copper chlorophyllin as a natural green food colorant, which can only be used to color dry citrus-fruit-based drink mixes [43].

Among the permitted carotenoid-based coloring agents, which produce color variations from yellow, orange to red, and violet, with more or less intense shades, there are as follows: carotenes (E160a), especially β-carotene, and annatto (E160b), containing the carotenoids bixin and norbixin as the main constituents; paprika extract, capsanthin, and capsorubin (E160c); lycopene (E160d); apocarotenal (E160e); lutein (E161b); and canthaxanthin (E161g) [171]. Of these, only β-carotene, paprika extract, capsanthin, and capsosubin are allowed as quantum satis. Canthaxanthin, for example, has a current acceptable daily intake of 0.03 mg/kg body weight [179]. These carotenoids are applied in the production of butter, margarines, oils, and fats, as well as in cheese spreads, jams, creams as well as jellies, pastries, rice, dairy products, flour, fish, soft drinks, meat products, sauces, marinades, seasoning mixtures, and others [17,171,180].

Currently, there is a wide variety of research where natural colorants have been incorporated into food products. For example, Obón et al. (2009) made a powdered colorant from Opuntia (*Opuntia stricta* Haw.) fruit juice, which was applied in food systems, namely yogurt and soft drink. The food products presented a vivid red–purple hue that was very attractive to consumers, which was maintained after a month under refrigeration (4 °C) [180]. In another study, the extraction of a carotenoid-rich colorant from tomato peel was optimized and the colorant was incorporated into a spaghetti formulation that had the highest score in sensory evaluation compared to the control and other samples [181]. Furthermore, the use of colorants in meat products is increasing. Coloring meat with paprika oleoresin or dried plum powder not only helps in its appearance, but also decreases oxidation processes, extending products’ shelf lives [182]. Likewise, the addition of an oily extract of chontaduro (*Bactris gasipaes* Kunth) residues is an alternative measure with which to reduce the use of nitriles in Frankfurter sausages, providing consumers with natural and healthy products [183].

In 2020, extracts of fig peels and blackthorn fruits were incorporated as natural purple colorants into donuts (icing) and into a typical Brazilian cake called “beijinho”, conceiving innovative products with natural pigments as well as antioxidant and antimicrobial properties [184]. Two studies carried out in Brazil succeeded in microencapsulating carotenoid compounds with high stability and controlling their release under specific conditions. The first one reported the stabilization of carotenoids from palm oil with chitosan/pectin and chitosan/xanthan by the complex coacervation method, where chitosan/xanthan microparticles showed the best potential for practical application in the food industry, especially in yogurt preparations. In the second study, authors microencapsulated purple Brazilian cherry juice (*Eugenia uniflora* L.) with high antioxidant potential, which was incorporated into yogurt and breads, demonstrating benefits to consumers [185,186].

## 6. Conclusion and Future Perspectives

In recent years, the importance of researching and increasing the use of natural colorants in novel and attractive food matrices has become evident. Pigments such as anthocyanins, carotenoids and chlorophylls not only add attractive colors to food products, but also provide consumers with therapeutic effects, such as antioxidant, antimicrobial, anticancer, and anti-inflammatory activities. Nevertheless, to make natural food colorants popular in the food industry, their high cost and low stability, as well as the strict regulations, standards, and lengthy toxicological evaluations by the FDA and the European Union, are bottlenecks that must be addressed through further investigation efforts. The recovery of pigments from food byproducts and residues by using clean technologies seems to be an irreversible tendency and surely the best way to make their production sustainable, although still being full of defiant aspects. Finally, future research should aim to broaden the information on the biochemical features of natural pigments, not only for creating strategies to solve their cost and stability issues but also to unravel their potential as functional food ingredients and nutraceuticals.

## Figures and Tables

**Table 1:** Studies on natural matrices rich in coloring compounds (anthocyanins, chlorophylls, and carotenoids) with their respective therapeutic potential.

Health-Promoting Effects	Matrix	Chemical Compound	Reference
Combat hyperglycemia and hyperuricemia	Cherries (*Prunus avium* L.) and purple sweet potato (*Ipomoea batatas* L.)	Anthocyanins	[59]
	Mulberry (*Morus alba* L.)		[60]
	Star gooseberry (*Sauropus androgynus* L.)	Chlorophylls	[42]
	Lettuce (*Lactuca sativa*)	Carotenoids	[61]
Anticancer	Blueberry (*Vaccinium myrtillus*)	Anthocyanins	[62]
	Black rice (*Oryza sativa* L. *indica*)		[63]
	Chokeberry (*Aronia meloncarpa* E.), elderberry (*Sambucus nigra* L.), bilberry (*Vaccinium myrtillus* L.), grape (*Vitis* *Vinifera* L.), purple carrot (*Daucus dacota* L.), purple corn (*Zea mays* L.), and red radish (*Raphanus sati* Vus L.)		[64]
	*Conyza trilova*	Chlorophylls	[65]
	Pomelo (*Citrus grandis*)		[41]
	Purple tomato (*Solanum lycopersicum* L. cv Micro-Tom)	Carotenoids	[66]
Cardiovascular disease	Elderberry (*S. nigra*), bilberry (*V. myrtillus*), and chokeberry (*A. melanocarpa*)	Anthocyanins	[67]
	Strawberry (*Fragaria × ananassa*) var. Alba		[68]
	Roselle (*Hibiscus sabdariffa* L.)		[69]
	Paprika (*Capsicum annuum*)	Carotenoids	[70]
Visual health	Bilberry (*Vaccinium myrtillus* L.)	Anthocyanins	[71]
	Seed coat of black soybean (*Glycine max* L.)		[72]
Antimicrobial	Ribes species, several cultivars (Ben Tirran, Lūšiai, Čiornyj negus, Corona’, Au Gs-5, and Jonkher van Tets)	Anthocyanins	[73]
	Mulberry (*Morus nigra* L.) and non-black mulberry (*Morus mongolica* and *Morus alba* L. ‘Zhenzhubai’)		[74]
	Mushrooms (*Lactarius deliciosus* (L.) Gray and *Lactarius piperatus* (L.) Pers)	Carotenoids	[75]
Antioxidant properties	Haskap (*Lonicera careulea* L.)	Anthocyanins	[25]
	Sweet cherry fruits (*Prunus avium* Linnaeus (L.))		[76]
	Broad-leaf bamboo (*Sasa senanensis*)	Chlorophylls	[77]
	Tomato (*Solanum lycopersicum* L.)	Carotenoids	[78]
	Carrot (*Daucus carota* L.) peels		[79]

**Table 3:** Comparison of different methods used for chlorophyll extraction.

Plant Matrix	Extraction Approach	Solvent	Conditions	Chlorophyll Recovery Yield	Reference
Sheets of Tifton 85 grass (*Cynodon* spp.)	Maceration	Dimethyl sulfoxide (DMSO)	Volume: 20 mL Eight evaluations of 12 h/12 h T (°C): 23–26 Humidity: 40–75%.	Chlorophyll *a*: 316 ± 2.93 µmol·m^−2^ Chlorophyll *b*: 66 ± 1.41 µmol·m^−2^	[111]
		N,N Dimethylformamide	Volume: 20 mL Eight evaluations of 12 h/12 h T (°C): 23–26 Humidity: 40–75%.	Chlorophyll *a*: 297 ± 3.58 µmol·m^−2^ Chlorophyll *b*: 85 ± 2.03 µmol·m^−2^	
		80% acetone	Volume: 20 mL Eight evaluations of 12 h/12 h T (°C): 23–26 Humidity: 40–75%.	Chlorophyll *a*: 250 ± 2.65 µmol·m^−2^ Chlorophyll *b*: 111 ± 1.50 µmol·m^−2^	
		Absolute ethanol	Volume: 20 mL Eight evaluations of 12 h/12 h T (°C): 23–26 Humidity: 40–75%.	Chlorophyll *a*: 259 ± 2.84 µmol·m^−2^ Chlorophyll *b*: 84 ± 2.25 µmol·m^−2^	
Sheets of canola (*Brassica napus* L. var *oleifera*)	Maceration	80% acetone	Conventional extraction	Chlorophyll *a*: 0.87 mg·g^−1^ Chlorophyll *b*: 0.39 mg·g^−1^	[117]
	No maceration	80% acetone	t (h): 24 Cold camera, no light	Chlorophyll *a*: 0.98 mg·g^−1^ Chlorophyll *b*: 0.38 mg·g^−1^	
Carrot (*Daucus carota* L.) and tomato (*Solanum lycopersicum* var. *cerasiforme*), aerial parts	Maceration	Ethanol/water 90/10 *v*/*v*	t (min): 60 and 120	Best: ethanol, 120 min Chlorophyll *a*: 2.46 ± 0.06 µg·g^−1^ Chlorophyll *b*: 28.5 ± 0.2 µg·g^−1^	[119]
	Ultrasound-assisted	Hexane	Power: 100, 200, and 400 W T (min): 5	Best: ethanol, 400 w Chlorophyll *a*: 107.7 ± 0.2 µg·g^−1^ Chlorophyll *b*: 99.6 ± 0.1 µg·g^−1^	
Microalgae (*Chlorella vulgaris*)	Maceration	Ethanol/water 90/10 *v*/*v*	T (°C): 30–60; t (h): 6–24 Solvent-to-biomass ratio: 20–90 mLsolv/g_biom_	Chlorophyll total: 53.47 mg·g^−1^ extr	[122]
Three hybrids, crosses between urucum (*Bixa* *orellana* L.)	Incubation Maceration	DMSO 80% acetone	T (°C): 25–65 t (h): 24, 48, and 72	DMSO for chlorophylls *a* and *b* > acetone 80% Acetone maximum point: 65 °C in 48 h	[123]
Chokecherry (*Prunus virginiana*) Alpines strawberry (*Fragaria vesca*) Sunflower (*Helianthus annuus*) Two graminoids (*Andropogon gerardii*, big bluestem; *Cymbopogon citrates*, lemongrass)	Maceration	DMSO 80% acetone	T (°C): 25, 30, and 40	Chl DMSO < acetone extraction for *C. citrates* Extraction efficiency was not influenced by temperature. The species may need to be macerated to extraction using DMSO	[85]
*R. capsulatus* CB1200 cultured in Tween 80, supplemented with growth medium	Maceration	Diethyl ether/ethanol (1:1)	Repeatedly washed with 20% ethanol	Chlorophyll *a*: 7 mg·L^−1^	[115]
*Chlorella vulgaris* (KMCC C-024)	Maceration (MAC)	Ethanol 90%	t (h): 6	Chlorophyll *a*: 4.26 ± 0.53 mg·g^−1^ sample Chlorophyll *b*: 2.58 ± 0.09 mg·g^−1^ sample	[120]
	Soxhlet (SOX)	Ethanol 90%	t (h): 2	Chlorophyll *a*: 3.32 ± 0.30 mg·g^−1^ sample Chlorophyll *b*: 3.45 ± 0.28 mg·g^−1^ sample	
	Ultrasound- assisted extraction (UAE)	Ethanol 90%	t (h): 2	Chlorophyll *a*: 5.12 ± 0.29 mg·g^−1^ sample Chlorophyll *b*: 3.71 ± 0.41 mg·g^−1^ sample	
	Pressurized liquid extraction (PLE)	Ethanol 90%	t (min): 8, 19, and 30 T (°C): 50, 105, and 160	Chlorophyll *a*: 9.63 ± 0.65 mg·g^−1^ sample Chlorophyll *b*: 5.77 ± 0.68 mg·g^−1^ sample	
Leaf pigments of two grapevine rootstock varieties (*Vitis vinifera* × *Vitis rotundifolia* and *Vitis riparia*)	Maceration	DMSO saturated with calcium carbonate	t (h): 24 and 48	Chlorophyll *a*: DMSO has been shown to be as efficient as that with 80% acetone Chlorophyll *b*: DMSO > acetone 80% for *V. vinifera* × *V. rotundifolia*	[116]
		Acetone 80%	t (h): 24 and 48		
*Clitoria fairchildiana* (Fabaceae) and *Gossypium* sp. (Malvaceae)	Maceration	Ethyl alcohol 95%	Room temperature for maceration and refrigeration for 48 h for conventional	*Clitoria fairchildiana* Maceration > conventional *Gossypium* sp. Without differentiation	[114]

**Table 5:** Advantages and disadvantages of some encapsulation methods and applications in different natural matrices rich in anthocyanins, carotenoids, and chlorophylls.

Encapsulation Method	Particle Size (μm)	Advantages	Disadvantages	Vegetable Source	Reference
Spray drying	10–100	Low process cost, fast, versatile, and the possibility of large-scale production in a continuous mode. High encapsulation efficiency and relatively good storage stability.	Degradation of temperature-sensitive compounds, non-uniform particulates, and small–moderate batch yields.	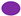 Haskap (*Lonicera careulea* L.)	[25]
				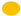 Pumpkin (*C. moschata*) peels	[156]
				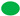 *Chlorella vulgaris*	[157]
Freeze-drying	20–5000	Possibility of encapsulating thermosensitive substances unstable in an aqueous solution.	Long times as well as high cost and energy. Low stability and sensitivity to oxidation.	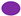 Jambolan (*Syzygium cumini* L.)	[158]
				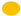 Carrot (*Daucus carota* L. cv. Heitianwucun)	[159]
				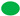 *Camellia sinensis* var. assamica	[146]
Fluidized bed	20–200	Low cost, specific capsule size distribution, low product porosity, and smooth as well as uniform drying method.	Drying sticky material is quite difficult. There is a possibility of fine product loss; chances of electrostatic build-up may be high.	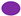 Blackberry (*Rubus fruticosus* L.) residue	[160]
				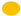 Carrot (*Daucus carota* L.)	[161]
				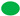 Turkey berries (*Solanum torvum* Swartz)	[162]
Emulsion polymerization	0.1–3	Micro–nanocapsules with a narrow size distribution.	Difficult to control the capsule formation (polymerization).	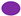 Blueberry (*Vaccinium augustifolium* Ait.) pomace	[151]
				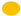 Ripe red bell peppers (*Capsicum annum* L.)	[152]
Ionic gelation	-	Low cost and does not require advanced equipment, high temperatures, and organic solvents.	Laboratory scale: capsules have a high porosity that favors intensive bursting.	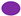 Hibiscus (*Hibiscus sabdariffa* L.)	[163]
				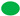 Stinging nettle (*Urtica urens* L.)	[164]
Thermal gelation	-	Uses gentle conditions, simple method.	Large gel porosity, low encapsulation efficiency.	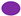 Blackberry fruits (*Rubus* spp.)	[147]
Phase separation (coacervation)	10–800	Ambient temperature, protection against oxidation and volatility, and the adapted release of active compounds.	High cost, complex, use of toxic chemicals, difficult to control particle size, and very sensitive to pH as well as ionic strength.	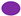 Blue barberry (*Berberis integerrima* Bunge)	[165]
				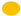 Commercial palm oil	[166]
				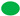 Fresh spinach (*Spinacia oleracea*)	[167]
Liposome entrapment	0.1–1	Can encapsulate aqueous or liposoluble material. Increased adsorption and bioavailability. Non-toxic and non-immunogenic.	Mainly used at the laboratory scale, unstable, expensive, and low encapsulation efficiency.	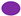 Black carrot	[168]
				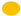 Annatto seeds (A-750-WS)	[169]
				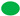 Leaves of *Chimonanthus salicifolius* S.Y.Hu	[170]

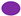
 Anthocyanins; 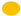 carotenoids; and 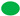 chlorophylls.

## Data Availability

The data presented in this study are available in this article.
